# Successful treatment with two siblings affected classic Bartter syndrome

**DOI:** 10.1186/1687-9856-2015-S1-P36

**Published:** 2015-04-28

**Authors:** Nguyen Ngoc Khanh, Vu Chi Dung

**Affiliations:** 1 National Hospital of Pediatrics, Hanoi, Vietnam

## 

Classic Bartter syndrome is a salt-wasting tubulopathy caused by mutations in the CLCNKB (chloride channel Kb) gene. Classic Bartter syndrome is characterized by early childhood onset. Herein, we report two Vietnamese siblings affected classic Bartter syndrome.

## Case presentation

Two siblings were admitted to National Hospital of Pediatrics with chief complaints of motor-development delay, growth retardation and failure of thrive. The 1^st^ child - a 39 month old boy with his birth weight of 1.9 kg presented with his height of 69 cm (-7.5 SD), his weight of 7.5 kg (-4.4 SD). The 2^nd^ child – an 18 months old girl presented with her height of 58 cm (-7.8 SD), her weight of 5.8 kg (-3.4 SD). They both developed polyuria (6ml/kg/hour), polydipsia, chronic dehydration and motor delay that they could not stand and walk but had normal intelligence. The investigations revealed hypokalemic metabolic alkalosis (PH: 7.5 – 7.51; pCO2: 45.9 - 56.8 mmHg; HCO3^-^: 36.6 - 45.7 mmol/l; serum potassium levels: 1.8 – 2.1 mmol/l), hyponatremia (125 – 128 mmol/l), hypochloremia (67 – 84 mmol/l), normal calcemia, and normal calciuria. They were treated with potassium supplement, indomethacin (2.5 mg/kg/day). After 15 months of treatment: height, weight of the 1^st^ boy and 2^nd^ girl were 94 cm (increasing 25cm; -3 SD), 12kg (increasing 4.5kg; -3 SD) and 84 cm (increasing 26 cm; -2.4 SD), 11 kg (increasing 5.2 kg; -1.3 SD), respectively. Their plasma electrolyte became normal after 2 weeks of treatment.

## Conclusions

Classic Bartter syndrome will have good prognosis if treated early. This is one cause that can result in growth retardation.

Written informed consent was obtained from the patient for publication of this Case report (and any accompanying images). A copy of the written consent is available for review by the Editor-in-Chief of this journal.

